# Application of Molecularly Imprinted Electrochemical Biomimetic Sensors for Detecting Small Molecule Food Contaminants

**DOI:** 10.3390/polym15010187

**Published:** 2022-12-30

**Authors:** Yunling Shao, Jiaqi Duan, Miao Wang, Jing Cao, Yongxin She, Zhen Cao, Guangyue Li, Fen Jin, Jing Wang, A. M. Abd El-Aty

**Affiliations:** 1 Institute of Quality Standardization & Testing Technology for Agro-Products, Chinese Academy of Agricultural Sciences, Beijing 100081, China; 2 Key Laboratory of Agrofood Safety and Quality (Beijing), Ministry of Agriculture and Rural Areas, Beijing 100081, China; 3 State Key Laboratory for Biology of Plant Diseases and Insect Pests, Institute of Plant Protection, Chinese Academy of Agricultural Sciences, Beijing 100081, China; 4 State Key Laboratory of Biobased Material and Green Papermaking, Shandong Academy of Sciences, Qilu University of Technology, Jinan 250353, China; 5 Department of Pharmacology, Faculty of Veterinary Medicine, Cairo University, Giza 12211, Egypt; 6 Department of Medical Pharmacology, Medical Faculty, Ataturk University, 25240 Erzurum, Turkey

**Keywords:** molecular imprinting, electrochemical biomimetic sensors, small-molecule chemical contaminants

## Abstract

Environmental chemical contaminants in food seriously impact human health and food safety. Successful detection methods can effectively monitor the potential risk of emerging chemical contaminants. Among them, molecularly imprinted polymers (MIPs) based on electrochemical biomimetic sensors overcome many drawbacks of conventional detection methods and offer opportunities to detect contaminants with simple equipment in an efficient, sensitive, and low-cost manner. We searched eligible papers through the Web of Science (2000–2022) and PubMed databases. Then, we introduced the sensing mechanism of MIPs, outlined the sample preparation methods, and summarized the MIP characterization and performance. The classification of electrochemistry, as well as its advantages and disadvantages, are also discussed. Furthermore, the representative application of MIP-based electrochemical biomimetic sensors for detecting small molecular chemical contaminants, such as antibiotics, pesticides, toxins, food additives, illegal additions, organic pollutants, and heavy metal ions in food, is demonstrated. Finally, the conclusions and future perspectives are summarized and discussed.

## 1. Introduction

Small molecule compounds, such as pesticides, veterinary drugs, mycotoxins, and environmental pollutants (persistent organic pollutants (POPs), dioxins, heavy metal ions), pose a risk to human health and pollute water, air, soil, and agricultural products [1,2]. Therefore, developing methods to monitor small molecule compounds is crucial. To detect small molecule compounds, a variety of methods, such as chromatography [3], chromatography-mass spectrometry [4], biological detection [5], and immunological approaches [6], have been developed. However, these methods have drawbacks and limitations, such as being time-consuming, requiring skilled labor, and restricting their use in situ and real-time detection [7].

In recent years, electrochemical sensors have been widely used to determine contaminants due to their sensitivity, rapid assay time, small size, portability, low cost, and low reagent content [8,9]. However, it is quite challenging to eliminate matrix interference while maintaining sensitivity [10]. To improve the sensitivity of electrochemical sensors, numerous techniques, such as nanomaterials, especially gold nanoparticles (AuNPs) [11,12], carbon nanotubes [13], and different electrode modifiers (ionic liquids and polymers) [14,15], have been used to improve the analytical performance in electrochemical sensors, which have demonstrated a suitable device for small molecule contaminant detection [6].

Biosensors include two main distinct components: a bioreceptor and a biorecognition element [16]. The biorecognition element is critical in determining the target analyte selectively and accurately. Antibodies bind specifically and selectively to their target antigens. However, making antibodies to recognize small molecule compounds is challenging because they have low molecular weights with a single antigenic determinant cluster. Furthermore, small molecule compounds are haptens with an acceptable reactogenicity profile; however, they are nonimmunogenic. For these reasons, designing compounds that *mimic antibodies* is greatly appreciated. Molecularly imprinted polymers (MIPs), as artificial antibodies, have offered a new option for the selective identification of target analytes [17]. MIPs are often called plastic antibodies, similar to naturally occurring antibodies [18]. They possess remarkable recognition properties that have been used in various applications, such as drug delivery, purification, and sensors [19,20,21]. Combining the advantages of MIPs and electrochemical sensors makes it possible to fabricate low-cost, convenient devices with high sensitivity and selectivity, quick response, superior chemical/mechanical stability, miniaturization, automation, reusability, and in situ detection of target analytes [22].

In this review, qualified studies were searched through the Web of Science (2000–2022) (http://www.webofscience.com/wos/alldb/basic-search, accessed on 1 October 2022) and PubMed databases (https://pubmed.ncbi.nlm.nih.gov/, accessed on 1 October 2022). To find appropriate literature, we combined the keyword phrase “electrochemical sensors” with the terms “molecularly imprinted technology,” “food contaminants”, “small-molecule chemical contaminants,” and “agro-food”. After evaluating the publication titles, keywords, and abstracts, valuable full-text articles were downloaded from the database. We demonstrate the MIP sensing mechanism in detail, summarize the preparation methods, and introduce the characterization and performance evaluation of MIPs. Second, electrochemical classification and its advantages and disadvantages are discussed. Moreover, we emphasize the application of MIP-based electrochemical biomimetic sensors for detecting antibiotic and pesticide residues, toxins, food additives, illegal additions, environmental organic pollutants (POPs), and heavy metal ions in food. Finally, the conclusions and prospects are discussed.

## 2. Molecular Imprinting Technology

### 2.1. The Principle of MIPs

**Figure 1:**
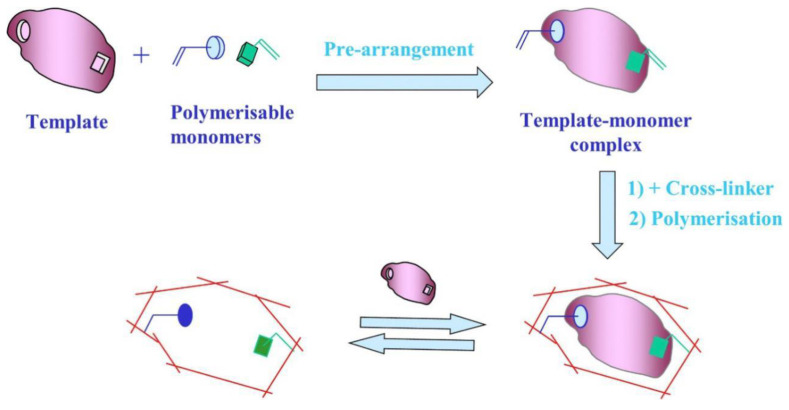
Preparation of MIPs. Adapted with permission from Ref. [24]. Copyright 2019, Elsevier.

Molecular imprinting technology (MIT) follows the “key and lock” principle for synthesizing polymers with specific recognition and selective adsorption to target molecules. These polymers are known as MIPs [23]. Although there are several production methods, they all follow the same basic pattern. The process generally includes three steps (Figure 1) [24]: (1) Under certain conditions, the template molecule and the functional monomer are self-assembled in a suitable solvent via reversible covalent, noncovalent, or semicovalent bonding between functional groups to form a template-monomer complex; (2) appropriate cross-linkers and initiators are added to the above system, and the other chemical bonds of the monomer interact with the cross-linkers through photopolymerization or thermal polymerization forming a network structure with a high degree of cross-linking and a particular three-dimensional space, allowing the functional groups to be fixed; and (3) finally, the template molecule is chemically or physically separated from the polymer, leaving matching three-dimensional cavities on the substrate’s surface. The stereo cavities in the imprinted layer serve a specific recognition function and can be selectively combined with templates from complex samples to achieve separation and detection [25]. The procedure is straightforward, quick, and convenient.

### 2.2. Preparation Methods

#### 2.2.1. Bulk Polymerization

Bulk or mass polymerization usually includes dissolving template molecules, functional monomers, crosslinkers, and initiators in a fixed ratio in solvents, such as chloroform, toluene, or acetonitrile, and then placing them in a glass or quartz vial to form a block polymer under light or thermal initiation, which is crushed and ground to obtain particles of appropriate size. Because of its simplicity and speed of preparation, bulk polymerization is the most convenient approach to synthesizing MIPs [26]. However, the grinding process creates an irregular morphology, which may result in considerable variation between different batches. Furthermore, some binding sites are destroyed, lowering extraction efficiency, selectivity, and reproducibility [27,28]. In addition, the technique requires many templates and is susceptible to template leakage and poor site accessibility. This is because the imprinted polymeric matrices are usually thick, and the residual template molecules and recognition sites are deeply embedded in the matrices, making them difficult to process [29,30]. Due to these factors, its applications and development are limited. The advantages and disadvantages of bulk polymerization are summarized in Table 1. 

#### 2.2.2. Suspension Polymerization

Suspension polymerization is a polymerization reaction that involves dispersing monomers into small droplets and suspending them in deionized water. The general reaction system is to add the organic phase (template molecules, functional monomers, crosslinkers, and initiators) to the aqueous phase or other strong polar solvents in which the dispersants are dissolved and then form a suspension by high-speed stirring. In this system, the dispersion forms uniform droplets under the shearing force of the water and protects the dispersant adsorbed on the surface. Then, the hydrophobic initiator triggers the polymerization of the monomers to obtain spherical molecularly imprinted polymers with a uniform particle size of approximately 10–100 μm. The particle size of the MIP can be used as a filler for HPLC and SPE due to the suspension method [30,31]. However, the suspension polymerization method adds the reaction components into the strong polar solvent, which can significantly interfere with the imprinting process by hydrogen bonding and weaken the binding between the template molecule and the functional monomer [32]. The advantages and disadvantages of suspension polymerization are summarized in Table 1.

#### 2.2.3. Emulsion Polymerization

Emulsion polymerization is similar to suspension polymerization, in which the template molecules, functional monomers, and crosslinkers are dissolved in the organic phase. Then, the organic mixture is transferred to the aqueous phase. After that, a stabilizer is added to the dispersed phase, preventing diffusion through the continuous phase and producing small, stable, uniformly sized emulsion droplets with particle sizes of approximately 50-1000 nm. The main advantages of this method are the high specific surface area, good dispersity of the prepared microspheres, narrow particle distribution, and ability to imprint water-soluble molecules [33]. The amount of emulsifier in this method can be adjusted to control the size of the polymer. Therefore, regular shapes and high yields of MIPs can be obtained [34]. This method produces high yields of monodisperse nanoparticles; however, the surfactant residues interfere with analyte identification during recombination, resulting in low binding capacity [35]. The advantages and disadvantages of emulsion polymerization are summarized in Table 1.

#### 2.2.4. Precipitation Polymerization

The suspension polymerization method dissolves the template molecules, functional monomers, crosslinkers, and initiators in the dispersant with a specific ratio and initiates polymerization with heat or light. The resulting polymer is saturated with the solvent, producing precipitation. The prepared imprinted polymerization particle size is uniform and small, with a microsphere size of approximately 0.2–2 μm [36]. The choice of functional monomer, solvent, and reactant ratio greatly influences the polymer yield and particle size. The precipitation polymerization method does not require the addition of stabilizers to the reaction system. The prepared polymers are uniformly distributed, the operation is straightforward with no complicated subsequent processing, and the utilization rate of raw materials and polymer yield is high. This method is characterized by a simple process, time savings, and high yield [37,38]. The major disadvantage is the strict requirement for solvent viscosity; the desired particle size can only be obtained in a solvent with a lower viscosity. The advantages and disadvantages of precipitation polymerization are summarized in Table 1.

#### 2.2.5. Surface Imprinting

The surface imprinting method causes the polymerization reaction to occur on the surface of the solid-phase carrier. It prepares a polymer with molecular imprinting recognition sites distributed on the surface of the solid-phase matrix [39]. The main advantages of this technology are as follows: the particle size of the prepared imprinted polymer is uniform and controllable by selecting the appropriate carrier, and the specific surface area of the imprinted polymer increases significantly when the carrier is a nanomaterial, which effectively improves the adsorption capacity and imprinting efficiency. Because the imprinted polymer is on the surface of the carrier, the encapsulation of the imprinted pores is effectively reduced. The imprinted polymer shell layer on the surface is relatively thin, so the adsorbed material transfers faster and can quickly reach the adsorption equilibrium state [25]. However, the surface area of the substrate is minimal, and accordingly, the total amount of the resultant imprinting cavities is always small [40]. Therefore, finding and preparing substrates with large surface areas is crucial for better imprinting performance. The advantages and disadvantages of surface imprinting are summarized in Table 1.

### 2.3. MIP Characterization Methods and Performance Evaluation

#### 2.3.1. MIP Characterization Methods

Scanning electron microscopy (SEM) and transmission electron microscopy (TEM) are commonly used to characterize the morphology of MIPs. SEM is essential for analyzing the surface morphology and pore characteristics of imprinted polymers [41]. TEM was used to observe the thickness of the shell layer of the polymer synthesized by the surface imprinting technique [42]. Atomic force microscopy (AFM) and various fluorescence techniques are crucial for characterizing thin-film MIPs [43]. Moreover, nuclear magnetic resonance (NMR) and Fourier transform infrared spectroscopy (FTIR) are used to analyze thin-film MIPs, which is becoming increasingly important. NMR is a powerful technique that can effectively verify the noncovalent bonding interplay between the template molecule and the functional monomer. FTIR can determine the structural changes of the template molecule in a solution or a solid-state [41,44]. If there is a hydrogen bonding interaction, then the positions of the peaks of the hydroxyl, carboxyl, or amino groups in the molecule will be shifted. X-ray derivatization (XRD) can determine whether there are crystallographic changes in the inorganic carrier [45]. If the thermal stability is examined, thermogravimetric analysis (TGA) can be used [45]. To synthesize core-shell polymers using surface imprinting techniques, TGA can also estimate the amount of grafting in the polymer shell [46]. For magnetic materials, such as Fe_3_O_4_, a vibrating sample magnetometer (VSM) is used to analyze the magnetic properties by plotting the hysteresis lines [47].

#### 2.3.2. MIP Performance Evaluation

##### Adsorption Isotherm Model

The equilibrium adsorption capacity is the most common parameter used to evaluate the performance of MIPs. This means the quantity of the target analytes adsorbed per unit mass of MIPs. To ensure equilibration, MIPs are exposed to the appropriate analyte in suitable solvents for a sufficiently long time [48,49]. The capacity is calculated as follows [48]:

(1) $$Q_{e}=\frac{(C_{0}-C_{e})V}{m}$$

where *C*_0_ (mg/mL) and *C_e_* (mg/mL) are the initial and equilibrium concentrations of the target analyte in the sample, respectively, *V* (mL) is the target analyte sample volume, and *m* (g) is the mass of MIPs or NIPs.

The Langmuir, Freundlich, and Scatchard models have been widely used for static adsorption equilibrium evaluation. The Langmuir model assumes that monolayer adsorption occurs in a homogeneous system and is expressed as follows:

(2) $$C_{e}/Q_{e}=C_{e}/Q_{m}+1/Q_{m}K_{L}$$

where *C_e_* (mg/mL) is the equilibrium concentration of targets, *Q_e_* (mg/g) and *Q_m_* (mg/g) are the equilibrium adsorption amounts and the maximum adsorption capacity of targets, respectively, and *K_L_* (mL/mg) is an affinity constant that is related to the affinity of the adsorbent for the binding sites [50].

The Freundlich model describes the adsorption of analytes on a heterogeneous surface of the sorbent, and it can be expressed as follows:

(3) $$\log Q_{e}=\log K_{F}+1/n\log C_{e}$$

where *K_F_* (mL/mg) and 1/*n* are the Freundlich characteristic constants and heterogeneity factor, respectively, and 1/*n* is often between 0 and 1, which shows the adsorption intensity of the target onto the adsorbent; the smaller the value, the more favorable the adsorption [51].

The Scatchard model, also known as the independent site-oriented adsorption model, helps to evaluate the binding properties and dependency of MIPs toward the analyte, and it can be estimated as follows:

(4) $$Q_{e}/C_{e}=\left(Q_{max}-Q_{e}\right)/K_{d}$$

where *Q_e_* (mg/g) is the adsorption capacity of the polymers at equilibrium, *Q_max_* is the maximum apparent adsorption capacity (mg/g), *C_e_* (mg/mL) is the equilibrium concentration of the target in solution, and *K_d_* is the equilibrium dissociation constant [48].

##### Adsorption Kinetics

During the dynamic adsorption equilibrium evaluation, the obtained data can be simulated and analyzed using pseudo-first-order kinetics, pseudo-second-order kinetics, and intraparticle diffusion models. The first two models are used to investigate the controlling mechanism, and the last is used for the diffusion mechanism. The pseudo-first-order model assumes that the diffusion step controls adsorption and can be evaluated as follows:

(5) $$\log(Q_{e}-Q_{t})=\log Q_{e}-k_{1}t$$

where *Q_e_* (mg/g) and *Q_t_* (mg/g) are the adsorption capacity at equilibrium time and at time t (min), respectively. *k*_1_ is the rate constant of the pseudo-first-order model [52].

The pseudo-second-order kinetic model is used to describe the chemisorption mechanism, which includes the sharing or exchange of electrons between the adsorbent and the ions to be enriched, and its equation is expressed as follows:

(6) $$t/Q_{t}=1/\left(k_{2}Q_{e}^{2}\right)+t/Q_{e}$$

where *k*_2_ is the rate constant of the pseudo-second-order model [52].

The above two kinetic models cannot describe the diffusion mechanism. Therefore, the intraparticle diffusion model has further studied the diffusion mechanism, and the equation is shown as follows:

(7) $$Q_{t}=k_{p}t_{1/2}+C$$

where *C* is the intercept and *k_p_* is the intraparticle diffusion rate constant, which can be obtained from the slope of the linear *Q_t_*~*t*_1/2_ [53].

##### Adsorption Selectivity

The selective adsorption properties of MIPs are commonly evaluated according to the imprinting factor (*IF*), which can be obtained as follows:

(8) $$IF=\frac{Q_{MIP}}{Q_{NIP}}$$

where *Q_MIP_* and *Q_NIP_* are the amounts of analyte bound by MIPs and NIPs, respectively [54].

The *MIP* polymer can distinguish the template and its analogs, which include the distribution coefficient (*K_d_*), selectivity coefficient (*k*), or relative selectivity coefficient (*K’*) [55].

(9) $$K_{d}=\frac{Q_{e}}{C_{e}}$$

where *Q_e_* (mg/g) is the adsorption capacity at equilibrium and *C_e_* (mg/mL) is the equilibrium concentration.

(10) $$K=\frac{K_{d1}}{K_{d2}}$$

where *K_d_*_1_ and *K_d_*_2_ are the analyte and analog distribution coefficients, respectively [56].

(11) $$K^{\prime }=\frac{K_{MIP}}{K_{NIP}}$$

where *K_MIP_* and *K_NIP_* are the MIP and NIP distribution coefficients, respectively.

##### Adsorption Performance

The solid phase extraction experiments are obtained via the following equation:

(12) $$E\%=\frac{C_{0}-C_{t}}{C_{0}}\times 100\%$$

where *C*_0_ (μg/L) and *C_t_* (μg/L) are the concentrations of the target before and after extraction, respectively [53].

Reuse time is another critical evaluation in practical industrial applications. To reduce cost, people hope that absorbents can be used multiple times instead of in a disposable manner [57].

##### Chromatographic Evaluation

Chromatographic evaluation is another method to describe the selectivity of molecularly imprinted polymers, and the retention factors of analytes were determined with MIPs and NIPs packed in the column, and they can be determined as follows:

(13) $$K=\frac{t_{R}-t_{0}}{t_{0}}$$

where *t_R_* (min) and *t*_0_ (min) are the analyte and unretained sample retention time in the column, respectively.

The *IF* is obtained by calculating the MIP and NIP columns’ capacity factor (*k*) ratio [18].

(14) $$IF=\frac{K_{MIP}}{K_{NIP}}$$

where *K_MIP_* and *K_NIP_* are the MIP and NIP distribution coefficients, respectively.

## 3. Electrochemical Sensors

**Figure 2:**
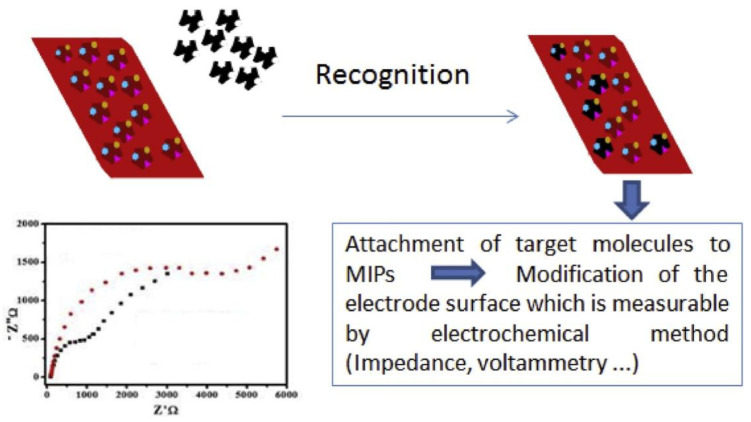
The principle of MIP-based electrochemical sensors. Adapted with permission from Ref. [58]. Copyright 2020, Elsevier.

Electrochemical sensors consist of two parts: receptors (recognition elements) and transducers (signal converts) [16]. In MIP electrochemical biomimetic sensors, the MIP membrane acts as a receptor and is immobilized on the transducer surface by an appropriate method. The principle of MIP-based electrochemical sensors is illustrated in Figure 2 [58]. When the target enters the specific cavity within the MIP membrane and binds specifically to its recognition site, the output electrical signal of the transducer changes. The detector can detect the signal for the determination of template molecules. Based on different response signals, sensors can be classified into current, potentiometry, capacitance, and conductivity [40].

### 3.1. Electric Current Sensors

Since Mosbach et al. [59] first constructed a MIP-based electric-current sensor, this technique has been widely reported. In MIP electric current sensors, quantitative analytes detect the current changes before and after template binding to MIPs. Since they are stable, sensitive, and selective, they are widely used [60].

**Figure 3:**
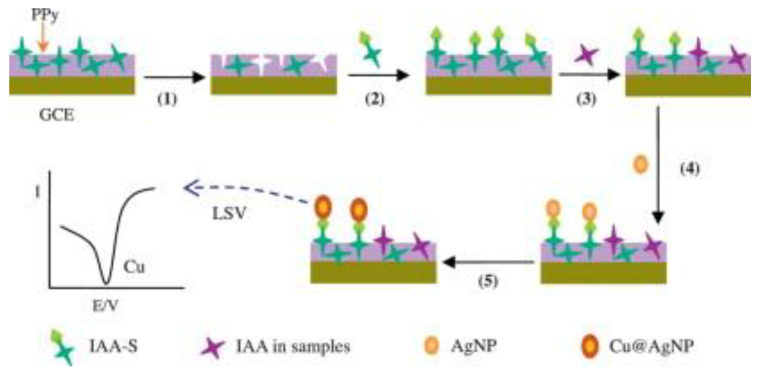
Schematic illustration of nonelectroactive molecular detection. (1) Elution; (2) IAA-S incubation; (3) IAA and labeled IAA-S competition; (4) AgNP labeling; and (5) catalytic copper deposition. Adapted with permission from Ref. [64]. Copyright 2014, Elsevier.

Amperometry and voltammetry are two main types of electric current sensors. Voltammetric techniques are most often applied, including differential pulse voltammetry (DPV), cyclic voltammetry (CV), square wave voltammetry (SWV), and linear sweep voltammetry (LSV) [61]. They can detect not only direct electroactive targets but also indirect nonelectroactive targets. The template molecule can penetrate the recognition holes in the imprinted membrane to reach the transducer surface and generate the corresponding electrical signal for the electroactive molecule. The quantitative analysis of the template molecules can be obtained by observing the magnitude of the electrical signal. Nonelectrically active molecules can be measured indirectly with the help of competitive measurements or the addition of special electrochemical signal probes [62,63]. When more template molecules occupy the recognition cavities in the imprinted membrane, there is less chance that the electrochemical probe can penetrate the imprinted membrane to reach the electrode surface, and the smaller the peak current of the electrochemical probe will be. For example, Li et al. proposed using a competitive measurement to recognize thiol-3-indoleacetic acid (IAA) [64]. Figure 3 depicts the detection procedure and principle for nonelectrically active molecules. The membrane is the key to electric current sensors; specific pores in the MIP membrane must exist so that the target molecules can penetrate through the membrane to the electrode surface. Since the MIP recognition ability is directly related to the MIP sensitivity, strategies for improving the MIP sensitivity are available here.

Merits: Simplicity, automation, miniaturization, high sensitivity, low cost, detection of electroactive and nonelectroactive molecules.

Demerits: Labeling the analyte to increase the electrochemical reaction at the working electrode.

### 3.2. Potentiometry Sensors

**Figure 4:**
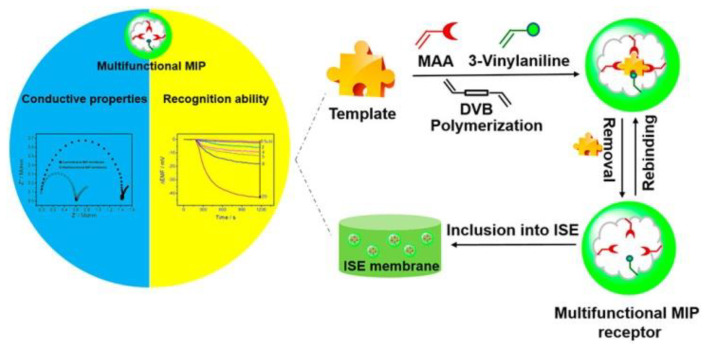
Schematic illustration of the MIP-based ISE sensor for neutral bisphenol detection using MIP for recognition on the ISE surface and charged ions as potential signals. Adapted with permission from Ref. [66]. Copyright 2022, American Chemical Society.

Potentiometric sensors measure the potential difference between the working electrode functionalized with MIPs and a reference electrode [65]. Compared with electric current sensors, potential signals are generated after target analytes bind to the imprinted membrane. Because the target analytes do not need to pass through the imprinted membrane, the imprinted template can be any size. Its combination with MIPs can substantially improve the selectivity of MIP potentiometric sensors. Potentiometry sensors are made up of two main components: ion-selective electrodes (ISEs) and field-effect transistors (FETs) [40]. ISEs are well known for ionic molecule selection, such as pH electrodes. MIP films are crucial in ISEs and have been used to detect ionic species. Selective membranes are formed from metal salts or polymeric (MIP) membranes containing ion exchangers or neutral carriers that can detect neutral molecules. For instance, Wang et al. proposed a novel MIP-based ISE sensor to detect neutral bisphenol with high selectivity [66]. The result exhibited high selectivity; Figure 4 schematically illustrates the process. In addition, field-effect transistors (FETs) are another semiconductor transducer sensitive to changes in surface potential at the gate electrode [67]. This device can practically monitor any charged template molecule. Potentiometric sensors are considered the most promising for use, independent of molecular size and rapid response; however, their stability and reproducibility are slightly poor.

Merits: Accessibility, high sensitivity, miniaturization, simplicity, low cost.

Demerits: Lack of specificity.

### 3.3. Capacitance/Impedance Sensors

Capacitance sensors, also called impedance sensors, are measured by detecting the imprinted membrane response to the template molecule capacitance before and after binding, providing an interfacial response signal without adding other reagents or probes, and are helpful for detecting nonelectroactive substances. The capacitance value of the capacitance sensor is determined by both the dielectric constant and the thickness of the electric layer, so it is necessary that the MIP-imprinted film fixed on the transducer surface has good insulation and is an ultrathin imprinted film. El-Akaad et al. developed a capacitive sensor based on MIPs that detects the insecticide imidacloprid in water. Electropolymerization showed satisfactory performance when the particles were immobilized on the surface of a gold electrode [68]. Capacitive sensors have the advantages of high sensitivity, label-free, real-time monitoring, and a simple manufacturing process. In addition, the film’s low thickness and high uniformity are the main advantages of capacitive sensors, and more work should be performed in future research.

Merits: Simplicity, cheap, fast, good sensitivity, biocompatibility with biological samples, no reference electrode, miniaturization.

Demerits: Low specificity and low sensitivity compared with amperometric and potentiometric methods.

### 3.4. Conductivity Sensors

Conductometric sensors measure conductivity variation before and after MIPs bind with target molecules [69]. The preparation of MIP films is an essential part of the development of conductivity sensors. Latif et al. prepared a conductive sensor for monitoring PAHs with MIP for recognition, and the sensor exhibited good performance [70]. This sensor is simple and inexpensive based on the electrical conductivity conversion principle. However, the synthesis and rinsing operations in the preparation process significantly affect the sensor performance, resulting in poor reproducibility and low sensitivity. These factors influence the broad use of conductometric sensors.

Merits: Label-free, simple, real-time monitoring, fast, inexpensive.

Demerits: Poor reproducibility.

## 4. Application of MIP Electrochemical Biomimetic Sensors for Detecting Small Molecule Chemical Food Contaminants

### 4.1. Antibiotic Residues

Antibiotics are extensively used to treat bacterial infections due to their broad spectrum of antibacterial activity. However, the incorrect use of antibiotics causes them to occur in water, food, and beverages. More seriously, the accumulation of antibiotics (parents and metabolites) due to misuse and overuse may result in antibiotic resistance [71,72]. Therefore, maximum residue limits (MRLs) have been set for antibiotics in food and the environment, and several analytical methodologies have been used to monitor antibiotics. Among these methods, MIP-based electrochemical techniques meet the requirements for detection [73].

**Figure 5:**
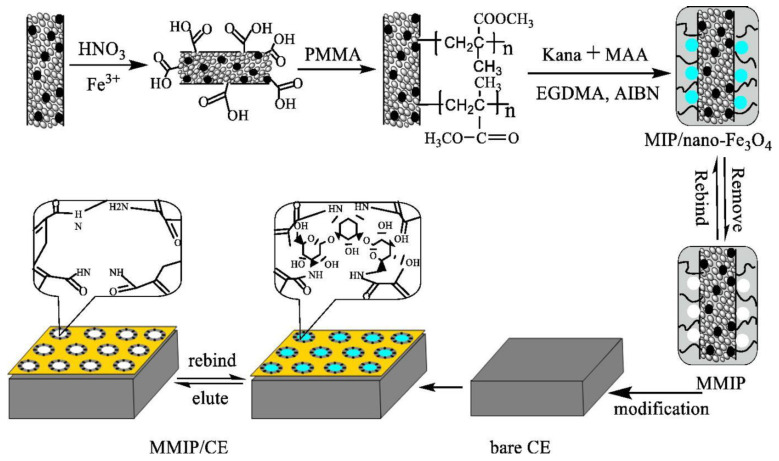
Schematic illustration of the magnetic imprinted electrochemical sensor. Adapted with permission from Ref [75]. Copyright 2015, Elsevier.

Antibiotics are divided into aminoglycosides, amphenicols, β-lactams, fluoroquinolones, macrolides, tetracyclines, and others based on their origin, structure, and mechanisms of microbial action [73,74]. Table 2 shows various methods developed to detect antibiotics based on MIP-electrochemical techniques. For instance, Long et al. fabricated a selective glass carbon electrode (GCE) based on MIP modified with magnetic multiwalled carbon nanotubes (MWCNTs) decorated with Fe_3_O_4_ for detecting kanamycin (Figure 5). The linear range was observed from 1.0 × 10^−10^ mol/L to 1.0 × 10^−6^ mol/L with a detection limit of 2.3 × 10^−11^ mol/L. The recoveries of kanamycin in real samples (chicken/liver, pig/liver, milk) ranged from 92.5–105.3%. The proposed imprinted sensor successfully used for kanamycin detection in complex real samples shows potential for consideration in the future [75]. The MIP-based electrochemical sensor indicated that it might avoid analog interference and improve detection efficiency. Erythromycin (Ery) is a macrolide that is extensively used in life. Ayankojo et al. prepared an electrochemical MIP-based sensor for Ery quantification using a screen-printed electrode (SPE). The MIP for Ery was constructed through the electropolymerization of m-phenylenediamine (mPD). CV was applied to detect the Ery bound to the MIP to prevent the template from oxidizing during testing. This sensor reached a LOD of 1.0 × 10^−10^ mol/L and was successfully applied to tap water. Moreover, Ery-SPE/MIP demonstrated good selectivity that can distinguish between target analytes and analogs [76].

### 4.2. Pesticide Residues

**Figure 6:**
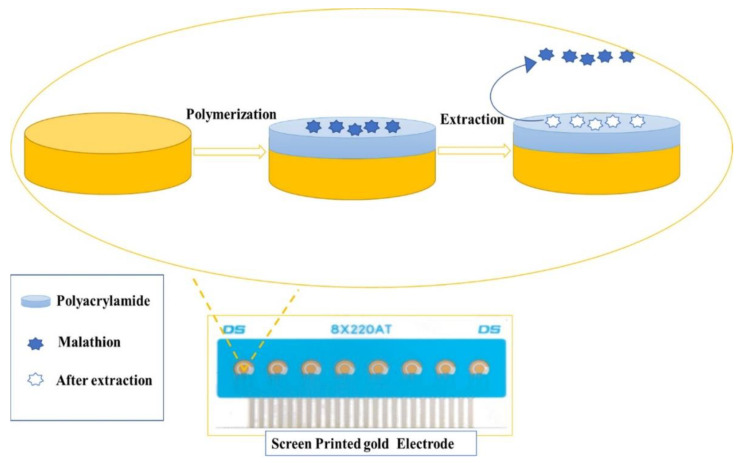
Schematic illustration of the experimental procedure of MIP-based SPE sensor fabrication. Adapted with permission from Ref. [94]. Copyright 2020, Elsevier.

Pesticides are used to prevent and combat different weeds, pests, or diseases to improve the quality of crops and production [31]. They are mostly sprayed on target plants or the soil. Notably, only a few pesticides are transmitted to target plants [89], and the rest accidentally reach the surface, the atmosphere, or underground waters. They can remain in the environment for a long time, causing serious concern [89,90]. Therefore, developing quick, sensitive, and reliable methods for quantitative pesticides is necessary. MIP-based electrochemical sensors are a valuable method used to monitor the detection of various pesticides. As shown in Table 3, the glass carbon electrode (GCE) and carbon paste electrode (CPE) are popular electrodes. For the detection of diazinon, a MIP-based CPE sensor was designed [91]. The MIPs were synthesized using diazinon as a template molecule and methacrylic acid (MAA) as a functional monomer. Cavities for diazinon were formed after the templates were removed. The CPE sensor and recognition of MIPs exhibited great sensitivity for diazinon and were successfully applied in water and apple fruit samples. Based on the same theory, MIPs combined with CPE sensors were used to detect hexazinone [92] and propazine [93]. Apart from GCE and CPE electrodes, screen-printed electrodes (SPEs) are also popularly used with MIPs [94]. For example, an electrochemical MIP sensor for the quantitative test of malathion has been devised (Figure 6). It was fabricated using an Au-SPE electrode by acrylamide polymerization in the presence of malathion as a template. The established method has proven to be highly accurate, rapid, and inexpensive for quantifying low levels of malathion residues in contaminated olive oil and fruit samples. To improve the sensitivity and magnify the sensor’s signal, nanoparticles (such as Au NPs and MWCNTs) are used for electrode modification. For example, Amatatongchai et al. prepared a sensor based on GCE electrodes coated with SiO_2_ and vinyl end groups to analyze profenofos (PFF). After the electrodeposition of MIP on the CNT/GCE surface, the electrode was immersed in a DMF solution. DPV could directly monitor the recognition by the MIP. The proposed sensor with high selectivity was successfully applied to determine PFF in vegetable samples [95].

### 4.3. Mycotoxins

Mycotoxins are a large and diverse group of naturally occurring chemicals mainly produced by strains of three fungal genera, namely, *Aspergillus*, *Penicillium,* and *Fusarium* [99]. Agricultural products are susceptible to mycotoxin contamination during harvest [100]. Although many countries have set and implemented MRLs, various food and agricultural products contaminated with mycotoxins still exceed the published guidelines and negatively influence humans and animals [101]. Therefore, effectively detecting trace amounts of mycotoxins in food samples is very valuable. Many researchers have noted the advantages of MIP-based sensors, which have been used for mycotoxin detection, as shown in Table 4.

**Figure 7:**
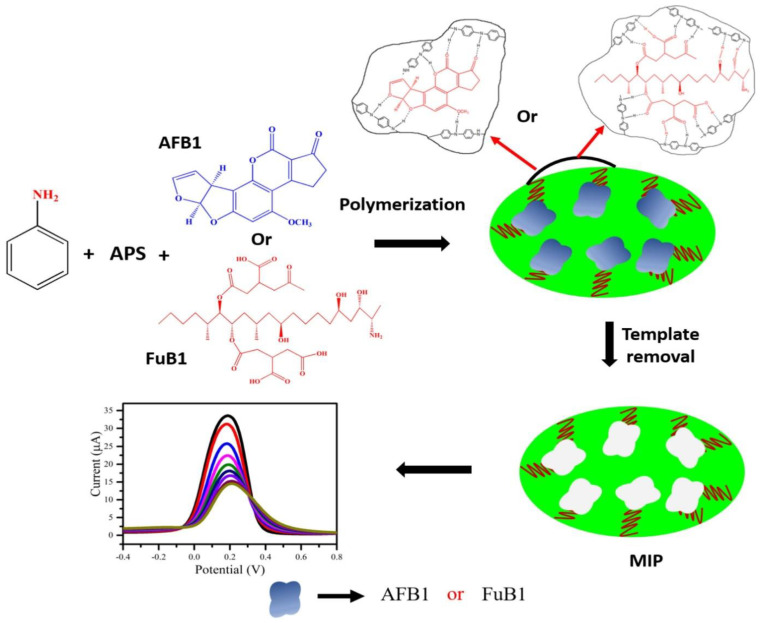
Schematic preparation of the MIP-based electrochemical sensing platform. Adapted with permission from Ref. [21]. Copyright 2021, Elsevier.

Singh et al. prepared an electrochemical sensing platform fabricated using MIP-based techniques for aflatoxin B1 (AFB1) and fumonisin B1 (FuB1) detection. During the MIP synthesis process, polyaniline was used as a MIP matrix, and AFB1 and FuB1 were used as template molecules (Figure 7). The proposed biosensors exhibited good sensitivity and low detection limits for AFB1 and FuB1, opening up a promising strategy to detect mycotoxins [21]. Radi et al. reported a MIP-based sensor for ZEA quantification using a screen-printed gold electrode SPGE modified with molecularly imprinted poly(o-phenylenediamine) (PPD) by electrosynthesis. The developed method was effectively applied to accurately determine ZEA in cornflakes and presented low LOD, excellent repeatability, and stability [102]. To increase sensitivity, Pacheco et al. used MWCNTs to fabricate a DPV sensor for ochratoxin A (OTA) detection in spiked beer and wine. The results indicated that the developed method is easy to operate and has the potential to be applied in the routine analysis of OTA in food samples [103]. Based on MIPs with electrochemical techniques, strategies can also be applied to detect deoxynivalenol in spiked beer and wine [104], and patulin in wheat flour [105].

### 4.4. Food Additives

Food additives are substances used in food to preserve flavor and improve taste, appearance, or other properties [106]. It is legally allowed to add a certain measure of food additives; however, the type of additives, the scope of use, the maximum amount of additives, and the residues are strictly regulated [107]. However, many food additives are inappropriately used in food for profit, and these substances are harmful to humans [107]. Various methods have been used to detect food additives in a sensitive, selective, and accurate manner. Analysts preferred MIP-based electrochemical sensors among them.

**Figure 8:**
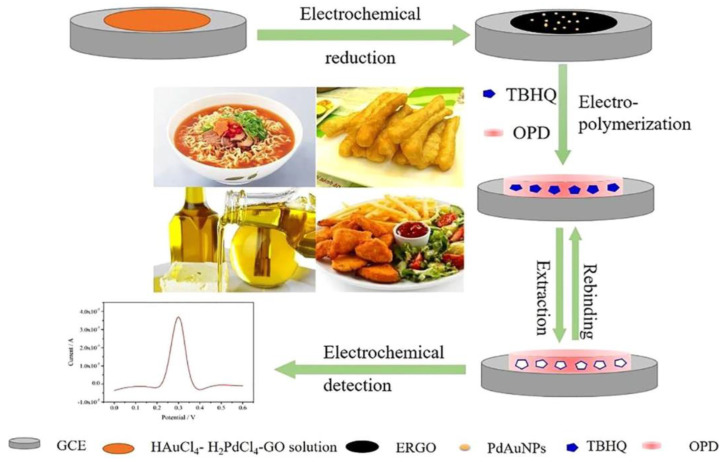
A schematic fabrication process of the MIP−based sensor for TBHQ detection. Adapted with permission from Ref. [109]. Copyright 2029, Elsevier.

Qin et al. developed a GCE sensor modified by graphene oxide (GO) decorated with Ag NPs [108]. Under optimal conditions, the proposed sensor has a wide range and a low limit of sunset yellow. This demonstrated that the sensor could be a reliable and straightforward method for practical sunset detection. The GO materials maximize the availability of the nanosized surface and provide fast mass transport to the binding sites. Another example is using a MIP-based sensor to detect butyl-hydroquinone (TBHQ) in spiked edible oil (Figure 8). The sensing phase of the sensor was built on the surface using MIPs, Pd Au nanoparticles, and reduced graphene oxide (GO). The sensor demonstrated good binding kinetics to TBHQ and high stability, selectivity, and sensitivity, with a LOD of 0.28 mol/L, and HPLC confirmed the results [109]. Some additives have a similar structure, such as sunset yellow and tartrazine. To distinguish them, Li et al. created an electrochemical sensor based on MIP to measure amaranth [110]. The sensor demonstrated a broad linear correlation range with low LOD and high recoveries, distinguishing amaranth from sunset yellow and tartrazine analogs, and was effectively used to assess amaranth in soft drinks.

### 4.5. Illegal Additives

Illegal food additives are nonfood substances prohibited in human food [111]. Melamine includes many nitrogen elements extensively employed in many fields, especially dairy products [112,113,114,115]. However, the illegal use of melamine in dairy products can harm humans and animals [115]. The U.S. Food and Drug Administration (FDA) and China’s Ministry of Health have stipulated melamine amounts [116]. Because of the advantages of MIPs coupled with electrochemical sensors, they are also used for melamine. Chen et al. prepared a facile sensor-based GCE modified with Au and polyaniline composite (Au@PANI) to amplify the sensor signal and increase the electrode. Then, the template melamine was further assembled onto Au@PANI. This sensor presents a simple but efficient low detection limit for melamine [117].

**Figure 9:**
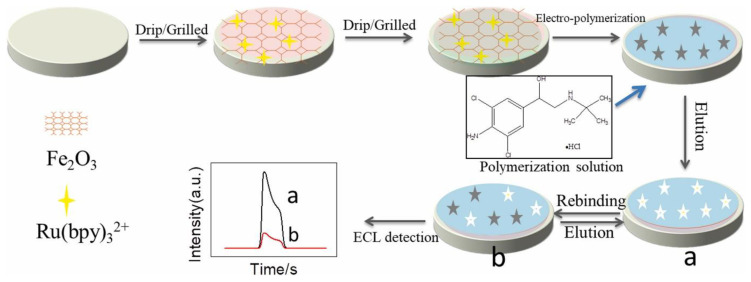
The different steps for the preparation of the sensor. Fe_2_O_3_ was used as the carrier to deposit Ru(bpy)_3_^2+^, and Fe_2_O_3_ @Ru(bpy)_3_^2+^ was used as a signal recognition layer. The ECL signal increased when the polymer was eluted from the MIPs (a), and the signal decreased (b) when the CLB rebinding to MIPs. Adapted with permission from Ref. [119]. Copyright 2022, Elsevier.

Clenbuterol is another illegal additive often used as a therapeutic drug for pulmonary disease. However, it is often misused in veterinary feeds to improve growth rates and increase lean muscle proportions [118]. To detect it rapidly and accurately, Zhao et al. [119] used clenbuterol hydrochloride (CLB) as the template molecule and pyrrole as the functional monomer to prepare MIPs on Fe_2_O_3_@Ru(bpy)_3_^2+^, and the prepared MIPs were applied to deposit the electrochemiluminescence (ECL) sensor. The fabrication steps of the ELC sensor are presented in Figure 9. The change in the ECL signal showed a linear standard curve with the concentration of CLB, and it showed low LOD and good recoveries, which can be used in practical life with high value.

### 4.6. Environmental Organic Pollutants

Persistent organic pollutants (POPs) are toxic and very persistent in soils, ranging from decades to centuries. They can be transported from local to global sources and bioaccumulate in the food chain, causing several health hazards and environmental effects [120]. POPs, which include PCDD/Fs, PCBs, and organochlorine pesticides (OCPs), are among the most significant and risky contaminants in soil [121].

**Figure 10:**
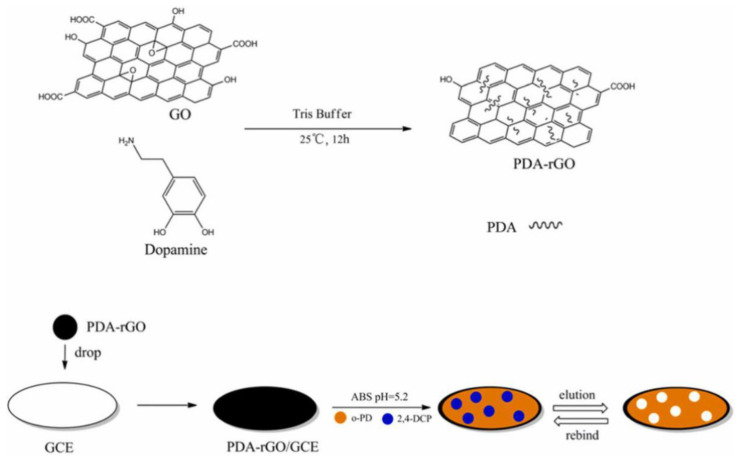
Schematic illustration of the electrochemical sensor for selective detection of 2,4-DCP. Adapted with permission from Ref. [122]. Copyright 2019, Elsevier.

PAHs (polycyclic aromatic hydrocarbons) are a class of organic pollutants that consist of at least two fused benzo rings. To detect them, Latif and colleagues synthesized MIPs based on a screen-printed interdigital gold electrode and used it as a conductometric sensor to determine PAHs [70]. The results from the conductive measurement showed that the sensor could detect PAHs with a LOD of 1.3 × 10^−9^ mol/L, which was selective and sensitive for anthracene detection in water. 2,4-Dichlorophenol (2,4-DCP) is a highly poisonous chlorophenol compound that has long-term effects on humans and animals. PDA-rGO was synthesized by Liu et al. and used as a supporting surface for the MIP (Figure 10). Based on this sensor, a specific and sensitive 2,4-DCP electrochemical sensor was developed and successfully applied to a water sample [122]. Since PCBs have no electrochemical activity, reports in the literature about electrochemical methods for detecting PCBs are limited. Beta-cyclodextrin (β-CD) has a special structure that can enable ferrocene to form host-guest inclusion complexes. PCB compounds can replace ferrocene in the cavity owing to their higher affinity toward β-CD. Based on this concept, an electrochemical sensor was built for the ultrasensitive detection of PCBs through a decrease in the ferrocene DPV signal, with a detection limit of 5 × 10^−13^ mol/L [123].

### 4.7. Heavy Metal Ions

Heavy metals are toxic and persistent chemical elements regardless of their concentration. Increased industrial activity and urbanization have led to heavy metal accumulation in soil and water sources. They can be transduced from local to global levels so that they may pose risks and hazards to humans and the ecosystem [124]. As shown in Table 5, some applications of electrochemical MIP-based sensors for monitoring metal ions in water and soil samples were observed. This section mainly introduces MIPs based on electrochemical sensors for common and toxic heavy metal ion detection, such as Pb^2+^, Hg^2+^, As^3+^, Cd^2+^, and Cr^3+^.

**Figure 11:**
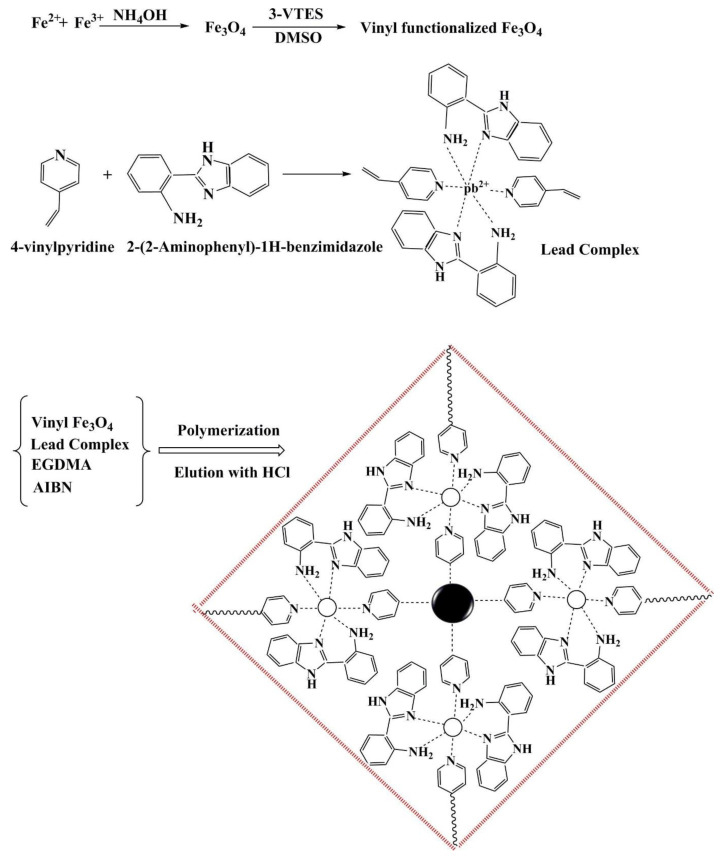
A schematic fabrication process of Fe_3_O_4_@SiO_2_@IIP. Adapted with permission from Ref. [125]. Copyright 2020, Elsevier.

Dahaghin et al. created a GPE with magnetic ion-imprinted nanoparticles Fe_3_O_4_@SiO_2_@IIP for efficient Pb^2+^ recognition in water and fruit juice [125]. For the synthesis of Fe_3_O_4_@SiO_2_@IIP, 4-vinyl pyridine was chosen as the functional monomer, and 2-(2-aminophenyl)-1H-benzimidazole was used as a binding ligand (Figure 11). The results showed that the developed sensor had excellent recognition behavior toward Pb^2+^ ions, with a low detection limit (2.4 × 10^−10^ mol/L) and a wide linear concentration range (4.8 × 10^−10^-3.5 × 10^−7^ mol/L). Another study by Alizadeh and colleagues reported a sensitive electrochemical sensor based on CPE coated with MWCNTs for detecting Hg^2+^ in environmental water samples [126]. It demonstrated acceptable sensing behavior toward the target Hg^2+^ ions over a linear concentration range of 1.0 × 10^−10^ mol/L to 2.0 × 10^−8^ mol/L, and the detection limit was 2.0 × 10^−10^ mol/L. Moreover, Ma et al. developed a gold electrode (GE) that was modified by an ion-imprinted polymer (IIP) and nanoporous gold (NPG) for As^3+^. The developed sensor demonstrated good reliability and specificity and was successfully applied to quantify As^3+^ in water [127]. Based on the same theory, different modification strategies are also used for Cd^2+^ [128] and Cr^3+^ [129].

## 5. Conclusions and Perspective

Small molecule chemical contaminants, such as mycotoxins, antibiotics, and pesticides, negatively influence human health and the environment. Therefore, developing rapid, accurate, and efficient analysis requirements is essential to detect these contaminants. Compared with traditional detection methods, electrochemical sensors overcome many limitations and promote efficiency, sensitivity, and low-cost detection with innovative miniaturized equipment. This view demonstrates the MIP sensing mechanism, summarizes the preparation methods, and introduces MIP characterization and performance evaluation. Second, electrochemical classification and its advantages and disadvantages are discussed. Moreover, we emphasize the application of MIP-based electrochemical biomimetic sensors for antibiotic residues, pesticide residues, toxins, food additives, illegal additions, environmental organic pollutants (POPs), and heavy metal ions. MIP-based electrochemical sensors for contaminant detection demonstrated a significant improvement.

Although MIPs have demonstrated their potential as recognition elements, the requirement for an extremely low LOD is still challenging. The sensitivity and affinity of MIPs are typically improved through nanomaterials, and strategies for improving MIP sensitivity are available here. This has inspired researchers to develop new and innovative MIP sensors for target molecule detection. Therefore, more nanomaterials and other new materials should be investigated in the future. There is no specific method for imprinting a specific class of molecules. As a result, the synthesis process and the functional monomers must be determined experimentally. Functional monomer investigation is also needed, and they may collaborate with other advanced technologies. For example, computational studies [118], reported in a few publications, are increasingly used to select suitable functional monomers.

## Figures and Tables

**Table 1:** The advantages and disadvantages of MIP preparation methods.

Preparation Methods	Advantages	Disadvantages	Ref.
Bulk polymerization	Simple, rapid, cheap, robust, resistant to harsh environments, and does not require a sophisticated or expensive analytical instrument.	Irregular morphology, low yield, template leakage, binding sites deeply buried, destroyed binding sites.	[26,27,28,29,30]
Suspension polymerization	Regular particles.	Poor recognition, polydisperse size, and polarity solvent interfere with the imprinting process.	[30,31,32]
Emulsion polymerization	High specific surface area, regular shape, size, good dispersity, narrow particle distribution, water-soluble.	Low binding capacity.	[33,34,35]
Precipitation polymerization	No stabilizers, simple, good yields, less time, small and uniform size, and suitable im-print of different compounds.	High dilution conditions, careful adjustment of the synthetic parameters, and a large porogen volume.	[36,37,38]
Surface imprinting	Uniform and controllable particle size, good selectivity and stability, high adsorption capacity, fast mass transfer and binding kinetics, and good reproducibility.	Limited surface areas.	[25,39,40]

**Table 2:** Detection of food antibiotic residues using different MIP electrochemical biomimetic sensors.

Class	Electrochemical Techniques	Functional Monomer	Target	Polymerization Method	Transducer (Modified)	Sample	LOD (mol/L)	Linear Range (mol/L)	Ref.
Aminoglycosides	CV	o-phenylenediamine	Kanamycin	Electropoly-merization	GCE-SWCNH-COOH	Water	1.0 × 10^−5^	1.0-5.0 × 10^−5^	[77]
	CV and DPV	MAA	Kanamycin	Surface imprinting	GCE-CNT (Fe_3_O_4_)	Chicken/liver, pig/liver, milk	2.3 × 10^−11^	1.0 × 10^−10^–1.0 × 10^−6^	[75]
	DPV	Pyrrole-3-carboxylic acid	Streptomycin	Electropolymerization	GCE (PPy3C/ERGO)	Porcine kidney, honey	0.5 × 10^−9^	0.2–8.0 × 10^−8^, 0.08–1.0 × 10^−6^	[78]
	SWV	o-phenylenediamine	Streptomycin	One-pot method	ITO	Milk, honey	1.72 × 10^−10^	8.6 × 10^−8^–3.44 × 10^−5^	[79]
Amphenicols	DPV	C_16_VimCl	Chloramphenicol	Surface imprinting	GCE (P-r-GO, CKM-3)	Milk, honey	1.0 × 10^−10^	5.0 × 10^−9^–5.0 × 10^−7^, 5.0 × 10^−7^–4.0 × 10^−6^	[80]
β-lactams	CV	Acrylamide	Amoxicillin	Bulk polymerization	SPE	Water	1.89 ± 1.03 × 10^−9^; 0.54 ± 0.1 × 10^−9^	0.01–5 × 10^−7^	[81]
	CV	o-phenylenediamine	Ampicillin	Electropolymerization	GCE (Au NPs/SWCNTs)	Milk	1.0 × 10^−9^	5.0 × 10^−8^–1.0 × 10^−5^	[82]
	DPV	MAA	Cloxacillin	Bulk polymerization	SPCE (GO-Au NPs)	Milk	3.6 × 10^−8^	1.1–7.5 × 10^−7^	[83]
Fluoroquinolones	CV	MAA	Ciprofloxacin	Bulk polymerization	GCE (Ch-AuNP)	Water, milk, pharmaceuticals	2.1 × 10^−7^	0.01–1 × 10^−4^	[43]
	CV and SWV	Pyrrole and o-phenylenediamine	Enrofloxacin	Electropolymerization	PGE	Pharmaceuticals	6.57 × 10^−13^	1.0 × 10^−4^–1.0 × 10^−10^	[84]
	CV	Pyrrole	Norfloxacin	Electropolymerization	GCE (CoFe-MOFs/Au NPs)	Milk	1.31 × 10^−13^	0.05–1.0 × 10^−10^, 0.1–1.0 × 10^−9^, 1.0–6.0 × 10^−9^	[85]
Macrolides	DPV	4-ABA	Azithromycin	Electropolymerization	SPCE	Water	8.0 × 10^−8^	0.05–1.0 × 10^−5^	[86]
	CV	m-phenylenediamine	Erythromycin	Electropolymerization	SPE	Water	1.0 × 10^−10^	0.2–1.6 × 10^−8^	[76]
Tetracyclines	CV	Dopamine and oligonucleotides	Tetracycline	Electropolymerization	GCE (Au NPs)	Milk	1.44 × 10^−13^	5.0 × 10^−9^–1.0 × 10^−7^, 1.0 × 10^−9^–1.0 × 10^−6^	[87]
	DPV	3-Aminopropyltriethoxysiloxane	Oxytetracycline	Surface imprinting polymerization	Magneto electrode	Milk	__	2.17 × 10^−9^–2.17 × 10^−4^	[88]

**Table 3:** Detection of food pesticide residues by different MIP electrochemical biomimetic sensors.

Electrochemical Techniques	Functional Monomer	Target	Polymerization Method	Transducer (Modified)	Sample	LOD (mol/L)	Linear Range (mol/L)	Ref.
CV and SWV	methylpropenoic acid	diazinon	Suspension polymerization	CPE	Well water, apple fruit	7.9 × 10^−10^	2.5 × 10^−9^–1.0 × 10^−7^, 1.0 × 10^−7^–2.0 × 10^−6^	[91]
DPV	2-vinylpyridine	hexazinone	Noncovalent approach	CPE	Water	2.6 × 10^−12^	1.9 × 10^−11^–1.1 × 10^−10^	[92]
DPV	acrylamide	propazine	Precipitation polymerization	CPE	Onion, tomato, lettuce	1.0 × 10^−9^	0.01–1.0 × 10^−6^, 0.1–5.5 × 10^−5^	[93]
CV and DPV	acrylamide	malathion	Deposition polymerization	SPE (Au NPs)	Olive oils, fruits	1.8 × 10^−11^	3.0 × 10^−13^–3 × 10^−9^	[94]
CV and DPV	Aminobenzoic acid	carbofuran	Electropolymerization	GCE (Au NPs)	Vegetable	2.4 × 10^−8^	5.0 × 10^−8^–4.0 × 10^−4^	[17]
DPV	methacrylic acid, vinyl benzene	chloridazon	Precipitation polymerization	CPE (MWCNT)	Water	6.2 × 10^−8^	5.7 × 10^−7^–4.0 × 10^−4^	[95]
SWV	MAA	diuron	Bulk polymerization	CPE (MWCNT-COOH)	Water	9.0 × 10^−9^	5.2 × 10^−8^–1.25 × 10^−6^	[96]
CV	MAA	methyl parathion	Precipitation polymerization	CPE	Soil, vegetable	3.4 × 10^−13^	1.0 × 10^−12^–8.0 × 10^−9^	[97]
CV	MAA	paraoxon	Surface imprinting polymerization	GCE (3D-CNTs)	Vegetable	2 × 10^−9^	1.010^−8^–2 × 10^−4^	[98]

**Table 4:** Detection of food mycotoxins by different MIP electrochemical biomimetic sensors.

Electrochemical Techniques	Functional Monomer	Target	Polymerization Method	Transducer (Modified)	Sample	LOD (mol/L)	Linear Range (mol/L)	Ref.
DPV	Aniline	AFB1, FuB1	Chemical oxidative polymerization	A–ITO, F–ITO	Corn	1.0 × 10^−12^ (AFB1), 4.6 × 10^−13^ (FuB1)	3.2 × 10^−12^–1.6 × 10^−9^ (AFB1), 1.4 × 10^−12^–7.0 × 10^−10^ (FuB1)	[21]
CV	o–phenylenediamine	Zearalenone	Electropolymerization	SPGE	Corn flakes	6.3 × 10^−10^	7.85 × 10^−9^–6.28 × 10^−7^	[102]
DPV	pyrrole	ochratoxin A	Electropolymerization	GCE (MWCNTs)	Spiked beer, wine	4.1 × 10^−9^	0.05–1.0 × 10^−6^	[103]
CV	L–arginine	deoxynivalenol	Electropolymerization	GCE (COOH–MWCNTs)	Wheat flour	7.0 × 10^−8^	1.0 × 10^−7^–7.0 × 10^−5^	[104]
DPV	aniline	patulin	Electropolymerization	GCE (Au@Cu–MOF/N–GQDs)	Apple juice	4.6 × 10^−12^	6.5 × 10^−12^–4.6 × 10^−7^	[105]

**Table 5:** Detection of heavy metal ions by different MIP electrochemical biomimetic sensors.

Electrochemical Techniques	Functional Monomer	Target	Polymerization Method	Electrode (Modified)	Sample	LOD (mol/L)	Linear Range (mol/L)	Ref.
CV	4–vinyl pyridine	Pb^2+^	Suspension polymerization	GCE	Water, fruit juice	2.4 × 10^−10^	4.8 × 10^−10^–3.5 × 10^−7^	[125]
SWV	Itaconic acid	Hg^2+^	Precipitation polymerization	CPE	Water	2.9 × 10^−11^	1.0 × 10^−10^–2.0 × 10^−8^	[126]
CV	o–phenylenediamine	AS^3+^	Electropolymerization	GE (IIP–NPG)	Water	7.1 × 10^−12^	2.0 × 10^−11^–9.0 × 10^−9^	[127]
DPV	MAA	Cd^2+^	Bulk polymerization	CPE (IIP)	Spiked water, rice, blood	1.99 × 10^−9^	4.0 × 10^−9^–5.0 × 10^−7^	[128]
ISEs	Itaconic acid	Cr^3+^	Thermal polymerization	CPE (IIP–MWCNTs)	Sea, river water, soil	5.9 × 10^−7^	1.0 × 10^−6^–1.0 × 10^−1^	[129]

## Data Availability

Not applicable.

## References

[1] Zhao F., Shi R., Liu R., Tian Y., Yang Z. (2021). Application of phage-display developed antibody and antigen substitutes in immunoassays for small molecule contaminants analysis: A mini-review. Food Chem..

[2] Binder E.M., Tan L.M., Chin L.J., Handl J., Richard J. (2007). Worldwide occurrence of mycotoxins in commodities, feeds and feed ingredients. Anim. Feed Sci. Technol..

[3] Liu C., Jiang Y.L., Xiu L.Y., Qian R.J., Zhao M.X., Luo P.J., Ke Y.B., Li G.M., Jiang W.X. (2021). Ultratrace Analysis of Neomycin Residues in Milk at Femtogram Levels by Flow-Through Immunoaffinity Chromatography Test. Food Anal. Methods.

[4] Vegh R., Soros C., Majercsik N., Sipos L. (2022). Determination of Pesticides in Bee Pollen: Validation of a Multiresidue High-Performance Liquid Chromatography-Mass Spectrometry/Mass Spectrometry Method and Testing Pollen Samples of Selected Botanical Origin. J. Agric. Food Chem..

[5] Carneiro S.V., Holanda M.H.B., Cunha H.O., Oliveira J.J.P., Pontes S.M.A., Cruz A.A.C., Fechine L.M.U.D., Moura T.A., Paschoal A.R., Zambelli R.A. (2021). Highly sensitive sensing of food additives based on fluorescent carbon quantum dots. J. Photoch. Photobio. A.

[6] Chen Z.J., Wu H.L., Shen Y.D., Wang H., Zhang Y.F., Hammock B., Li Z.F., Luo L., Lei H.T., Xu Z.L. (2022). Phosphate-triggered ratiometric fluoroimmunoassay based on nanobody-alkaline phosphatase fusion for sensitive detection of 1-naphthol for the exposure assessment of pesticide carbaryl. J. Hazard. Mater..

[7] Rasheed T., Bilal M., Nabeel F., Adeel M., Iqbal H.M.N. (2019). Environmentally-related contaminants of high concern: Potential sources and analytical modalities for detection, quantification, and treatment. Environ. Int..

[8] Cetinkaya A., Kaya S.I., Atici E.B., Corman M.E., Uzun L., Ozkan S.A. (2022). A semi-covalent molecularly imprinted electrochemical sensor for rapid and selective detection of tiotropium bromide. Anal. Bioanal. Chem..

[9] Bakhshpour M., Göktürk I., Gür S.D., Yılmaz F., Denizli A., Siddiqui S., Meghvansi M.K., Chaudhary K.K. (2022). Sensor Applications for Detection in Agricultural Products, Foods, and Water. Pesticides Bioremediation.

[10] Venkatalaxmi A., Padmavathi B.S., Amaranath T. (2004). A general solution of unsteady Stokes equations. FlDyR.

[11] Cheng W., Zhang Q., Wu D., Yang Y., Zhang Y., Tang X. (2022). A facile electrochemical method for rapid determination of 3-chloropropane-1,2-diol in soy sauce based on nanoporous gold capped with molecularly imprinted polymer. Food Control.

[12] Abu Shama N., Asir S., Ozsoz M., Gokturk I., Turkmen D., Yilmaz F., Denizli A. (2022). Gold-Modified Molecularly Imprinted N-Methacryloyl-(l)-phenylalanine-containing Electrodes for Electrochemical Detection of Dopamine. Bioengineering.

[13] Demir E., Inam O., Inam R. (2018). Determination of Ophthalmic Drug Proparacaine Using Multi-walled Carbon Nanotube Paste Electrode by Square Wave Stripping Voltammetry. Anal. Sci..

[14] Chen Y., Tang Y., Liu Y., Zhao F., Zeng B. (2022). Kill two birds with one stone: Selective and fast removal and sensitive determination of oxytetracycline using surface molecularly imprinted polymer based on ionic liquid and ATRP polymerization. J. Hazard. Mater..

[15] Rahman S., Bozal-Palabiyik B., Unal D.N., Erkmen C., Siddiq M., Shah A., Uslu B. (2022). Molecularly imprinted polymers (MIPs) combined with nanomaterials as electrochemical sensing applications for environmental pollutants. Trends Environ. Anal. Chem..

[16] Velusamy V., Arshak K., Korostynska O., Oliwa K., Adley C. (2010). An overview of foodborne pathogen detection: In the perspective of biosensors. Biotechnol. Adv..

[17] Qi P., Wang J., Wang X., Wang Z., Xu H., Di S., Wang Q., Wang X. (2018). Sensitive and selective detection of the highly toxic pesticide carbofuran in vegetable samples by a molecularly imprinted electrochemical sensor with signal enhancement by AuNPs. RSC Adv..

[18] Azizi A., Bottaro C.S. (2020). A critical review of molecularly imprinted polymers for the analysis of organic pollutants in environmental water samples. J. Chromatogr. A.

[19] Medina Rangel P.X., Moroni E., Merlier F., Gheber L.A., Vago R., Tse Sum Bui B., Haupt K. (2020). Chemical Antibody Mimics Inhibit Cadherin-Mediated Cell-Cell Adhesion: A Promising Strategy for Cancer Therapy. Angew. Chem. Int. Ed. Engl..

[20] Wang W.R., Wang X.X., Cheng N., Luo Y.B., Lin Y.H., Xu W.T., Du D. (2020). Recent advances in nanomaterials-based electrochemical (bio)sensors for pesticides detection. TrAC Trend Anal. Chem..

[21] Singh A.K., Lakshmi G.B.V.S., Fernandes M., Sarkar T., Gulati P., Singh R.P., Solanki P.R. (2021). A simple detection platform based on molecularly imprinted polymer for AFB1 and FuB1 mycotoxins. Microchem. J..

[22] Rebelo P., Costa-Rama E., Seguro I., Pacheco J.G., Nouws H.P.A., Cordeiro M., Delerue-Matos C. (2021). Molecularly imprinted polymer-based electrochemical sensors for environmental analysis. Biosens. Bioelectron..

[23] Carballido L., Karbowiak T., Cayot P., Gerometta M., Sok N., Bou-Maroun E. (2022). Applications of molecularly imprinted polymers and perspectives for their use as food quality trackers. Chem-Us.

[24] Turiel E., Martín-Esteban A. (2019). Molecularly imprinted polymers-based microextraction techniques. TrAC Trends Anal. Chem..

[25] Dong C., Shi H., Han Y., Yang Y., Wang R., Men J. (2021). Molecularly imprinted polymers by the surface imprinting technique. Eur. Polym. J..

[26] Ashley J., Shahbazi M.A., Kant K., Chidambara V.A., Wolff A., Bang D.D., Sun Y. (2017). Molecularly imprinted polymers for sample preparation and biosensing in food analysis: Progress and perspectives. Biosens. Bioelectron..

[27] Zhang W., She X., Wang L., Fan H., Zhou Q., Huang X., Tang J.Z. (2017). Preparation, Characterization and Application of a Molecularly Imprinted Polymer for Selective Recognition of Sulpiride. Materials.

[28] Poliwoda A., Mościpan M., Wieczorek P.P. (2016). Application of Molecular Imprinted Polymers for Selective Solid Phase Extraction of Bisphenol A. Ecol. Chem. Eng. S.

[29] Zhao W., Sheng N., Zhu R., Wei F., Cai Z., Zhai M., Du S., Hu Q. (2010). Preparation of dummy template imprinted polymers at surface of silica microparticles for the selective extraction of trace bisphenol A from water samples. J. Hazard. Mater..

[30] Song Z.H., Li J.H., Lu W.H., Li B.W., Yang G.Q., Bi Y., Arabi M., Wang X.Y., Ma J.P., Chen L.X. (2022). Molecularly imprinted polymers based materials and their applications in chromatographic and electrophoretic separations. TrAC Trend Anal. Chem..

[31] Demir Ö., Ulusoy H.İ., Özer E.T., Osman B. (2020). Development of a new solid phase extraction method for sensitive determination of some carbamate pesticides in water using poly(EGDMA-MATrp) microbeads. Microchem. J..

[32] Zhou X., Lai C., Huang D., Zeng G., Chen L., Qin L., Xu P., Cheng M., Huang C., Zhang C. (2018). Preparation of water-compatible molecularly imprinted thiol-functionalized activated titanium dioxide: Selective adsorption and efficient photodegradation of 2, 4-dinitrophenol in aqueous solution. J. Hazard. Mater..

[33] Wang Z.H., Qiu T., Guo L.H., Ye J., He L.F., Li X.Y. (2017). The synthesis of hydrophilic molecularly imprinted polymer microspheres and their application for selective removal of bisphenol A from water. React. Funct. Polym..

[34] Yang J., Li Y., Wang J., Sun X., Cao R., Sun H., Huang C., Chen J. (2015). Molecularly imprinted polymer microspheres prepared by Pickering emulsion polymerization for selective solid-phase extraction of eight bisphenols from human urine samples. Anal. Chim. Acta.

[35] Zhou T.Y., Ding L., Che G.B., Jiang W., Sang L. (2019). Recent advances and trends of molecularly imprinted polymers for specific recognition in aqueous matrix: Preparation and application in sample pretreatment. TrAC Trend Anal. Chem..

[36] Pardeshi S., Singh S.K. (2016). Precipitation polymerization: A versatile tool for preparing molecularly imprinted polymer beads for chromatography applications. RSC Adv..

[37] Zeng H., Yu X., Wan J., Cao X. (2020). Rational design and synthesis of molecularly imprinted polymers (MIP) for purifying tylosin by seeded precipitation polymerization. Process Biochem..

[38] Lai J.P., Yang M.L., Niessner R., Knopp D. (2007). Molecularly imprinted microspheres and nanospheres for di(2-ethylhexyl)phthalate prepared by precipitation polymerization. Anal. Bioanal. Chem..

[39] Eersels K., Lieberzeit P., Wagner P. (2016). A review on synthetic receptors for bio-particle detection created by surface-imprinting techniques—From principles to applications. ACS Sens..

[40] Chen L., Wang X., Lu W., Wu X., Li J. (2016). Molecular imprinting: Perspectives and applications. Chem. Soc. Rev..

[41] Arias P.G., Martinez-Perez-Cejuela H., Combes A., Pichon V., Pereira E., Herrero-Martinez J.M., Bravo M. (2020). Selective solid-phase extraction of organophosphorus pesticides and their oxon-derivatives from water samples using molecularly imprinted polymer followed by high-performance liquid chromatography with UV detection. J. Chromatogr. A.

[42] Tian L., Guo H., Li J., Yan L., Zhu E., Liu X., Li K. (2021). Fabrication of a near-infrared excitation surface molecular imprinting ratiometric fluorescent probe for sensitive and rapid detecting perfluorooctane sulfonate in complex matrix. J. Hazard. Mater..

[43] Surya S.G., Khatoon S., Ait Lahcen A., Nguyen A.T.H., Dzantiev B.B., Tarannum N., Salama K.N. (2020). A chitosan gold nanoparticles molecularly imprinted polymer based ciprofloxacin sensor. RSC Adv..

[44] Moein M.M. (2021). Advancements of chiral molecularly imprinted polymers in separation and sensor fields: A review of the last decade. Talanta.

[45] Li L., Zheng X., Chi Y., Wang Y., Sun X., Yue Q., Gao B., Xu S. (2020). Molecularly imprinted carbon nanosheets supported TiO2: Strong selectivity and synergic adsorption-photocatalysis for antibiotics removal. J. Hazard. Mater..

[46] Yang Z., Wang J., Shah T., Liu P., Ahmad M., Zhang Q., Zhang B. (2021). Development of surface imprinted heterogeneous nitrogen-doped magnetic carbon nanotubes as promising materials for protein separation and purification. Talanta.

[47] Zhang K.Y., Wang Y.F., Wen Q.Y., Huang Q.R., Li T.Q., Zhang Y., Luo D.L. (2022). Preparation and characterization of magnetic molecularly imprinted polymer for specific adsorption of wheat gliadin. J. Mol. Struct..

[48] Feng G., Sun J., Wang M., Wang M., Li Z., Wang S., Zheng L., Wang J., She Y., Abd El-Aty A.M. (2021). Preparation of molecularly imprinted polymer with class-specific recognition for determination of 29 sulfonylurea herbicides in agro-products. J. Chromatogr. A.

[49] Benedetti B., Di Carro M., Magi E. (2019). Multivariate optimization of an extraction procedure based on magnetic molecular imprinted polymer for the determination of polycyclic aromatic hydrocarbons in sea water. Microchem. J..

[50] Wang M., Liang S., Bai L., Qiao F., Yan H. (2019). Green protocol for the preparation of hydrophilic molecularly imprinted resin in water for the efficient selective extraction and determination of plant hormones from bean sprouts. Anal. Chim. Acta.

[51] Hu W., Xie Y., Lu S., Li P., Xie T., Zhang Y., Wang Y. (2019). One-step synthesis of nitrogen-doped sludge carbon as a bifunctional material for the adsorption and catalytic oxidation of organic pollutants. Sci. Total Environ..

[52] Zhu G., Cheng G., Lu T., Cao Z., Wang L., Li Q., Fan J. (2019). An ionic liquid functionalized polymer for simultaneous removal of four phenolic pollutants in real environmental samples. J. Hazard. Mater..

[53] Liang W., Lu Y., Li N., Li H., Zhu F. (2020). Microwave-assisted synthesis of magnetic surface molecular imprinted polymer for adsorption and solid phase extraction of 4-nitrophenol in wastewater. Microchem. J..

[54] Wang P., Zhu H., Liu J., Ma Y., Yao J., Dai X., Pan J. (2019). Double affinity integrated MIPs nanoparticles for specific separation of glycoproteins: A combination of synergistic multiple bindings and imprinting effect. Chem. Eng. J..

[55] Ndunda E.N. (2020). Molecularly imprinted polymers—A closer look at the control polymer used in determining the imprinting effect: A mini review. J. Mol. Recognit..

[56] Yu X., Zeng H., Wan J., Cao X. (2020). Computational design of a molecularly imprinted polymer compatible with an aqueous environment for solid phase extraction of chenodeoxycholic acid. J. Chromatogr. A.

[57] Zhang Y., Qin L., Cui Y., Liu W.-f., Liu X.-g., Yang Y.-z. (2020). A hydrophilic surface molecularly imprinted polymer on a spherical porous carbon support for selective phenol removal from coking wastewater. New Carbon Mater..

[58] Zouaoui F., Bourouina-Bacha S., Bourouina M., Jaffrezic-Renault N., Zine N., Errachid A. (2020). Electrochemical sensors based on molecularly imprinted chitosan: A review. TrAC Trend Anal. Chem..

[59] Kriz D., Mosbach K. (1995). Competitive Amperometric Morphine Sensor-Based on an Agarose Immobilized Molecularly Imprinted Polymer. Anal. Chim. Acta.

[60] Wang W. (2022). Electrochemical sensor based on molecularly imprinted membranes at Au@CNTs nanocomposite-modified electrode for determination of prednisolone as a doping agent in sport. Int. J. Electrochem. Sci..

[61] Elfadil D., Lamaoui A., Della Pelle F., Amine A., Compagnone D. (2021). Molecularly Imprinted Polymers Combined with Electrochemical Sensors for Food Contaminants Analysis. Molecules.

[62] Richter E.M., Rocha D.P., Cardoso R.M., Keefe E.M., Foster C.W., Munoz R.A.A., Banks C.E. (2019). Complete Additively Manufactured (3D-Printed) Electrochemical Sensing Platform. Anal. Chem..

[63] Beluomini M.A., da Silva J.L., de Sa A.C., Buffon E., Pereira T.C., Stradiotto N.R. (2019). Electrochemical sensors based on molecularly imprinted polymer on nanostructured carbon materials: A review. J. Electroanal. Chem..

[64] Li J.P., Yin W.L., Tan Y.J., Pan H.C. (2014). A sensitive electrochemical molecularly imprinted sensor based on catalytic amplification by silver nanoparticles for 3-indoleacetic acid determination. Sens. Actuators B.

[65] Thevenot D.R., Toth K., Durst R.A., Wilson G.S. (2001). Electrochemical biosensors: Recommended definitions and classification. Biosens. Bioelectron..

[66] Wang C., Qi L., Liang R., Qin W. (2022). Multifunctional Molecularly Imprinted Receptor-Based Polymeric Membrane Potentiometric Sensor for Sensitive Detection of Bisphenol A. Anal. Chem..

[67] Iskierko Z., Checinska A., Sharma P.S., Golebiewska K., Noworyta K., Borowicz P., Fronc K., Bandi V., D’Souza F., Kutner W. (2017). Molecularly imprinted polymer based extended-gate field-effect transistor chemosensors for phenylalanine enantioselective sensing. J. Mater. Chem. C.

[68] El-Akaad S., Mohamed M.A., Abdelwahab N.S., Abdelaleem E.A., De Saeger S., Beloglazova N. (2020). Capacitive sensor based on molecularly imprinted polymers for detection of the insecticide imidacloprid in water. Sci. Rep..

[69] Pohanka M., Skládal P. (2008). Electrochemical biosensors—Principles and applications. J. Appl. Biomed..

[70] Latif U., Ping L., Dickert F.L. (2018). Conductometric Sensor for PAH Detection with Molecularly Imprinted Polymer as Recognition Layer. Sensors.

[71] Honeychurch K.C., Piano M. (2022). Sensors for Environmental Monitoring and Food Safety. Biosensors.

[72] Tarannum N., Khatoon S., Dzantiev B.B. (2020). Perspective and application of molecular imprinting approach for antibiotic detection in food and environmental samples: A critical review. Food Control.

[73] de Faria L.V., Lisboa T.P., Campos N.D.S., Alves G.F., Matos M.A.C., Matos R.C., Munoz R.A.A. (2021). Electrochemical methods for the determination of antibiotic residues in milk: A critical review. Anal. Chim. Acta.

[74] Kemper N. (2008). Veterinary antibiotics in the aquatic and terrestrial environment. Ecol. Indic..

[75] Long F., Zhang Z., Yang Z., Zeng J., Jiang Y. (2015). Imprinted electrochemical sensor based on magnetic multi-walled carbon nanotube for sensitive determination of kanamycin. J. Electroanal. Chem..

[76] Ayankojo A.G., Reut J., Ciocan V., Opik A., Syritski V. (2020). Molecularly imprinted polymer-based sensor for electrochemical detection of erythromycin. Talanta.

[77] Han S., Li B.Q., Song Z., Pan S.H., Zhang Z.C., Yao H., Zhu S.Y., Xu G.B. (2017). A kanamycin sensor based on an electrosynthesized molecularly imprinted poly-o-phenylenediamine film on a single-walled carbon nanohorn modified glassy carbon electrode. Analyst.

[78] Wen Y., Liao X., Deng C., Liu G., Yan Q., Li L., Wang X. (2017). Imprinted voltammetric streptomycin sensor based on a glassy carbon electrode modified with electropolymerized poly(pyrrole-3-carboxy acid) and electrochemically reduced graphene oxide. Microchim. Acta.

[79] Liu B., Tang D., Zhang B., Que X., Yang H., Chen G. (2013). Au(III)-promoted magnetic molecularly imprinted polymer nanospheres for electrochemical determination of streptomycin residues in food. Biosens. Bioelectron..

[80] Yang G., Zhao F. (2015). Electrochemical sensor for chloramphenicol based on novel multiwalled carbon nanotubes@molecularly imprinted polymer. Biosens. Bioelectron..

[81] Jamieson O., Soares T.C.C., de Faria B.A., Hudson A., Mecozzi F., Rowley-Neale S.J., Banks C.E., Gruber J., Novakovic K., Peeters M. (2019). Screen Printed Electrode Based Detection Systems for the Antibiotic Amoxicillin in Aqueous Samples Utilising Molecularly Imprinted Polymers as Synthetic Receptors. Chemosensors.

[82] Shi X., Ren X., Jing N., Zhang J. (2020). Electrochemical Determination of Ampicillin Based on an Electropolymerized Poly(o-Phenylenediamine)/Gold Nanoparticle/Single-Walled Carbon Nanotube Modified Glassy Carbon Electrode. Anal. Lett..

[83] Jafari S., Dehghani M., Nasirizadeh N., Baghersad M.H., Azimzadeh M. (2019). Label-free electrochemical detection of Cloxacillin antibiotic in milk samples based on molecularly imprinted polymer and graphene oxide-gold nanocomposite. Measurement.

[84] Yan C., Zhang R., Chen Y., Wang G. (2017). Electrochemical determination of enrofloxacin based on molecularly imprinted polymer via one-step electro-copolymerization of pyrrole and o -phenylenediamine. J. Electroanal. Chem..

[85] Ye C., Chen X., Zhang D., Xu J., Xi H., Wu T., Deng D., Xiong C., Zhang J., Huang G. (2021). Study on the properties and reaction mechanism of polypyrrole@norfloxacin molecularly imprinted electrochemical sensor based on three-dimensional CoFe-MOFs/AuNPs. Electrochim. Acta.

[86] Rebelo P., Pacheco J.G., Cordeiro M.N.D.S., Melo A., Delerue-Matos C. (2020). Azithromycin electrochemical detection using a molecularly imprinted polymer prepared on a disposable screen-printed electrode. Anal. Methods.

[87] Rad A.O., Azadbakht A. (2019). An aptamer embedded in a molecularly imprinted polymer for impedimetric determination of tetracycline. Microchim. Acta.

[88] Yang Y., Shi Z., Chang Y., Wang X., Yu L., Guo C., Zhang J., Bai B., Sun D., Fan S. (2021). Surface molecularly imprinted magnetic MOFs: A novel platform coupled with magneto electrode for high throughput electrochemical sensing analysis of oxytetracycline in foods. Food Chem..

[89] Reynoso E.C., Torres E., Bettazzi F., Palchetti I. (2019). Trends and Perspectives in Immunosensors for Determination of Currently-Used Pesticides: The Case of Glyphosate, Organophosphates, and Neonicotinoids. Biosensors.

[90] Kumar P., Kim K.H., Deep A. (2015). Recent advancements in sensing techniques based on functional materials for organophosphate pesticides. Biosens. Bioelectron..

[91] Motaharian A., Motaharian F., Abnous K., Hosseini M.R., Hassanzadeh-Khayyat M. (2016). Molecularly imprinted polymer nanoparticles-based electrochemical sensor for determination of diazinon pesticide in well water and apple fruit samples. Anal. Bioanal. Chem..

[92] Toro M.J.U., Marestoni L.D., Sotomayor M.D.P.T. (2015). A new biomimetic sensor based on molecularly imprinted polymers for highly sensitive and selective determination of hexazinone herbicide. Sens. Actuators B.

[93] Gholivand M.B., Karimian N., Malekzadeh G. (2012). Computational design and synthesis of a high selective molecularly imprinted polymer for voltammetric sensing of propazine in food samples. Talanta.

[94] Aghoutane Y., Diouf A., Osterlund L., Bouchikhi B., El Bari N. (2020). Development of a molecularly imprinted polymer electrochemical sensor and its application for sensitive detection and determination of malathion in olive fruits and oils. Bioelectrochemistry.

[95] Ghorbani A. (2020). Detection of Chloridazon in Aqueous Matrices Using a Nano- Sized Chloridazon-Imprinted Polymer-Based Voltammetric Sensor. Int. J. Electrochem. Sci..

[96] Wong A., Foguel M.V., Khan S., Oliveira F.M.d., Tarley C.R.T., Sotomayor M.D.P.T. (2015). Development of an Electrochemical Sensor Modified with Mwcnt-Cooh and Mip for Detection of Diuron. Electrochim. Acta.

[97] Li Y., Liu J., Zhang Y., Gu M., Wang D., Dang Y.Y., Ye B.C., Li Y. (2018). A robust electrochemical sensing platform using carbon paste electrode modified with molecularly imprinted microsphere and its application on methyl parathion detection. Biosens. Bioelectron..

[98] Amatatongchai M., Sroysee W., Sodkrathok P., Kesangam N., Chairam S., Jarujamrus P. (2019). Novel three-Dimensional molecularly imprinted polymer-coated carbon nanotubes (3D-CNTs@MIP) for selective detection of profenofos in food. Anal. Chim. Acta.

[99] Mupunga I., Lebelo S.L., Mngqawa P., Rheeder J.P., Katerere D.R. (2014). Natural occurrence of aflatoxins in peanuts and peanut butter from Bulawayo, Zimbabwe. J. Food Prot..

[100] Lee H.J., Ryu D. (2017). Worldwide Occurrence of Mycotoxins in Cereals and Cereal-Derived Food Products: Public Health Perspectives of Their Co-occurrence. J. Agric. Food Chem..

[101] Luo S., Du H., Kebede H., Liu Y., Xing F. (2021). Contamination status of major mycotoxins in agricultural product and food stuff in Europe. Food Control.

[102] Radi A.E., Eissa A., Wahdan T. (2020). Molecularly Imprinted Impedimetric Sensor for Determination of Mycotoxin Zearalenone. Electroanalysis.

[103] Pacheco J.G., Castro M., Machado S., Barroso M.F., Nouws H.P.A., Delerue-Matos C. (2015). Molecularly imprinted electrochemical sensor for ochratoxin A detection in food samples. Sens. Actuators B.

[104] Li W., Diao K., Qiu D., Zeng Y., Tang K., Zhu Y., Sheng Y., Wen Y., Li M. (2021). A highly-sensitive and selective antibody-like sensor based on molecularly imprinted poly(L-arginine) on COOH-MWCNTs for electrochemical recognition and detection of deoxynivalenol. Food Chem..

[105] Hatamluyi B., Rezayi M., Beheshti H.R., Boroushaki M.T. (2020). Ultra-sensitive molecularly imprinted electrochemical sensor for patulin detection based on a novel assembling strategy using Au@Cu-MOF/N-GQDs. Sens. Actuators B.

[106] Ishidate M., Sofuni T., Yoshikawa K., Hayashi M., Nohmi T., Sawada M., Matsuoka A. (1984). Primary mutagenicity screening of food additives currently used in Japan. Food Chem. Toxicol..

[107] Carocho M., Morales P., Ferreira I.C.F.R. (2015). Natural food additives: Quo vadis?. Trends Food Sci. Technol..

[108] Qin C., Guo W., Liu Y., Liu Z., Qiu J., Peng J. (2017). A Novel Electrochemical Sensor Based on Graphene Oxide Decorated with Silver Nanoparticles–Molecular Imprinted Polymers for Determination of Sunset Yellow in Soft Drinks. Food Anal. Methods.

[109] Yue X., Luo X., Zhou Z., Bai Y. (2019). Selective electrochemical determination of tertiary butylhydroquinone in edible oils based on an in-situ assembly molecularly imprinted polymer sensor. Food Chem..

[110] Li L., Zheng H., Guo L., Qu L., Yu L. (2019). A sensitive and selective molecularly imprinted electrochemical sensor based on Pd-Cu bimetallic alloy functionalized graphene for detection of amaranth in soft drink. Talanta.

[111] Xiao D., Jiang Y., Bi Y. (2018). Molecularly imprinted polymers for the detection of illegal drugs and additives: A review. Microchim. Acta.

[112] Fu C., Liu C., Li Y., Guo Y., Luo F., Wang P., Guo L., Qiu B., Lin Z. (2016). Homogeneous Electrochemical Biosensor for Melamine Based on DNA Triplex Structure and Exonuclease III-Assisted Recycling Amplification. Anal. Chem..

[113] Giroto A.S., Garcia R.H.S., Colnago L.A., Klamczynski A., Glenn G.M., Ribeiro C. (2020). Role of urea and melamine as synergic co-plasticizers for starch composites for fertilizer application. Int. J. Biol. Macromol..

[114] Rovina K., Siddiquee S. (2016). Electrochemical sensor based rapid determination of melamine using ionic liquid/zinc oxide nanoparticles/chitosan/gold electrode. Food Control.

[115] Yu C., Li L., Ding Y., Liu H., Cui H., Zhang F., Lin J., Duan Y. (2021). A sensitive molecularly imprinted electrochemical aptasensor for highly specific determination of melamine. Food Chem..

[116] Chen X.Y., Ha W., Shi Y.P. (2019). Sensitive colorimetric detection of melamine in processed raw milk using asymmetrically PEGylated gold nanoparticles. Talanta.

[117] Rao H., Chen M., Ge H., Lu Z., Liu X., Zou P., Wang X., He H., Zeng X., Wang Y. (2017). A novel electrochemical sensor based on Au@PANI composites film modified glassy carbon electrode binding molecular imprinting technique for the determination of melamine. Biosens. Bioelectron..

[118] Zhang B., Fan X., Zhao D. (2018). Computer-Aided Design of Molecularly Imprinted Polymers for Simultaneous Detection of Clenbuterol and Its Metabolites. Polymers.

[119] Zhao Y., Tian L., Zhang X., Sun Z., Shan X., Wu Q., Chen R., Lu J. (2022). A novel molecularly imprinted polymer electrochemiluminescence sensor based on Fe_2_O_3_@Ru(bpy)32+ for determination of clenbuterol. Sens. Actuators B.

[120] Sinkkonen S., Paasivirta J. (2000). Degradation half-life times of PCDDs, PCDFs and PCBs for environmental fate modeling. Chemosphere.

[121] Weber R., Bell L., Watson A., Petrlik J., Paun M.C., Vijgen J. (2019). Assessment of pops contaminated sites and the need for stringent soil standards for food safety for the protection of human health. Environ. Pollut..

[122] Liu Y., Liang Y., Yang R., Li J., Qu L. (2019). A highly sensitive and selective electrochemical sensor based on polydopamine functionalized graphene and molecularly imprinted polymer for the 2,4-dichlorophenol recognition and detection. Talanta.

[123] Zheng X., Li H., Xia F., Tian D., Hua X., Qiao X., Zhou C. (2016). An Electrochemical Sensor for Ultrasensitive Determination the Polychlorinated Biphenyls. Electrochim. Acta.

[124] McLaughlin M.J., Hamon R.E., McLaren R.G., Speir T.W., Rogers S.L. (2000). Review: A bioavailability-based rationale for controlling metal and metalloid contamination of agricultural land in Australia and New Zealand. Aust. J. Soil Res..

[125] Dahaghin Z., Kilmartin P.A., Mousavi H.Z. (2020). Novel ion imprinted polymer electrochemical sensor for the selective detection of lead(II). Food Chem..

[126] Alizadeh T., Hamidi N., Ganjali M.R., Rafiei F. (2017). Determination of subnanomolar levels of mercury (II) by using a graphite paste electrode modified with MWCNTs and Hg(II)-imprinted polymer nanoparticles. Microchim. Acta.

[127] Ma W., Chang Q., Zhao J., Ye B.C. (2020). Novel electrochemical sensing platform based on ion imprinted polymer with nanoporous gold for ultrasensitive and selective determination of As(3). Microchim. Acta.

[128] Samandari L., Bahrami A., Shamsipur M., Farzin L., Hashemi B. (2019). Electrochemical preconcentration of ultra-trace Cd2+ from environmental and biological samples prior to its determination using carbon paste electrode impregnated with ion imprinted polymer nanoparticles. Int. J. Environ. Anal. Chem..

[129] Alizadeh T., Mirzaee S., Rafiei F. (2017). All-solid-state Cr(III)-selective potentiometric sensor based on Cr(III)-imprinted polymer nanomaterial/MWCNTs/carbon nanocomposite electrode. Int. J. Environ. Anal. Chem..

